# (*Z*)-2-{2,4-Dimeth­oxy-6-[(*E*)-4-meth­oxy­styr­yl]benzyl­idene}quinuclidin-3-one

**DOI:** 10.1107/S1600536812005843

**Published:** 2012-02-17

**Authors:** Nikhil Reddy Madadi, Sean Parkin, Peter A. Crooks

**Affiliations:** a Department of Pharmaceutical Sciences, College of Pharmacy, University of Kentucky, Lexington, KY 40536, USA; b Department of Chemistry, University of Kentucky, Lexington, KY 40506, USA; c Department of Pharmaceutical Sciences, College of Pharmacy, University of Arkansas for Medical Sciences, Little Rock, AR 72205, USA

## Abstract

The crystal structure of the title compound, C_25_H_27_NO_4_, shows the presence of a double bond with *Z* geometry which connects the quinuclidin-3-one ring and the trimeth­oxy­resveratrol moiety. The dihedral angle between the two benzene rings in the stilbene skeleton is 32.80 (8)°.

## Related literature

For related biological activity literature, see: Aggarwal *et al.* (2004 ▶); Pettit *et al.* (1995 ▶). For related structure–activity studies, see: Cushman *et al.* (1991 ▶). For related pharmaco­kinetic and pharmacodynamic studies, see: Jeandet *et al.* (1979 ▶); Trela *et al.* (1996 ▶).
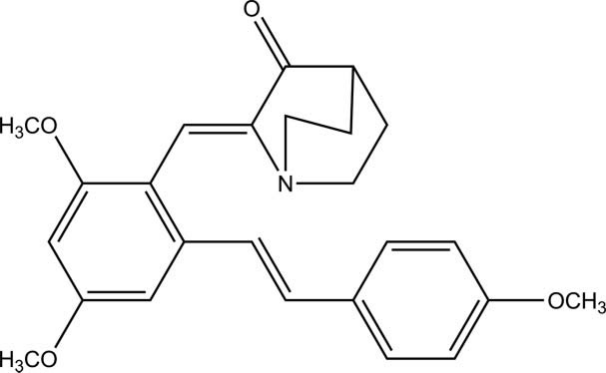


## Experimental

### 

#### Crystal data

C_25_H_27_NO_4_
*M* *_r_* = 405.48
Orthorhombic, 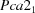

*a* = 36.1068 (1) Å
*b* = 6.8748 (1) Å
*c* = 8.4813 (4) Å
*V* = 2105.29 (10) Å^3^
*Z* = 4
Mo *K*α radiation
μ = 0.09 mm^−1^
*T* = 90 K
0.26 × 0.20 × 0.08 mm

#### Data collection

Nonius KappaCCD diffractometer
Absorption correction: multi-scan (*SCALEPACK*; Otwinowski & Minor, 1997 ▶) *T*_min_ = 0.978, *T*_max_ = 0.993
39211 measured reflections
2554 independent reflections
2313 reflections with *I* > 2σ(*I*)
*R*_int_ = 0.051

#### Refinement

*R*[*F* ^2^ > 2σ(*F* ^2^)] = 0.035
*wR*(*F* ^2^) = 0.090
*S* = 1.04
2554 reflections
274 parameters
1 restraint
H-atom parameters constrained
Δρ_max_ = 0.17 e Å^−3^
Δρ_min_ = −0.19 e Å^−3^

### 

Data collection: *COLLECT* (Nonius, 1998 ▶); cell refinement: *SCALEPACK* (Otwinowski & Minor, 1997 ▶); data reduction: *DENZO-SMN* (Otwinowski & Minor, 1997 ▶); program(s) used to solve structure: *SHELXS97* (Sheldrick, 2008 ▶); program(s) used to refine structure: *SHELXL97* (Sheldrick, 2008 ▶); molecular graphics: *XP* in *SHELXTL* (Sheldrick, 2008 ▶); software used to prepare material for publication: *SHELXL97* and local procedures.

## Supplementary Material

Crystal structure: contains datablock(s) global, I. DOI: 10.1107/S1600536812005843/fj2476sup1.cif

Structure factors: contains datablock(s) I. DOI: 10.1107/S1600536812005843/fj2476Isup2.hkl

Supplementary material file. DOI: 10.1107/S1600536812005843/fj2476Isup3.cml

Additional supplementary materials:  crystallographic information; 3D view; checkCIF report

## Figures and Tables

**Fig. 1.**
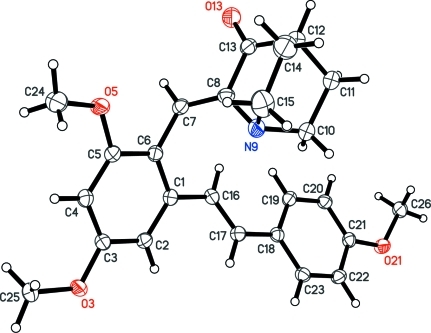
A view of the molecule with the atom numbering scheme. Displacement ellipsoids are drawn at the 50% probability level.

## supplementary crystallographic information

### Comment

Resveratrol (*trans*-3,4',5-trihydroxystilbene) is a phytochemical which is found in more than 70 plant species. This phytolaxine was proven to have diverse biological beneficial activities with no adverse effects in animal models (Aggarwal *et al.* 2004, Pettit *et al.* 1995). Resveratrol was also reported to be a potential cancer chemotherapeutic agent based on its striking inhibitory effects on cellular events associated with cancer initiation, promotion, and progression (Cushman *et al.* 1991). Unfortunately, resveratrol cannot be used as a drug because of its chemical and metabolic instability (Jeandet *et al.* 1979, Trela *et al.* 1996). *trans*-3,4',5-trimethoxystilbene, an analog of resveratrol, was found to have greater chemical and metabolic stability with improved anticancer activity. Based on several SAR studies on trimethoxyresveratrol analogues we have designed and synthesized a series of novel trimethoxy resveratrol analogues that are expected to function as potent cytotoxic agents against breast and lung cancer cells. The X-ray analysis of the titled compound was performed to determine the geometry (*i.e. E versus Z*) of the double bond connecting the quinuclidin-3-one ring and the trimethoxyresveratrol moiety, and to obtain detailed information on the structural conformation of the molecule that may be useful in structure-activity relationship (SAR) analysis. The title compound was synthesized in two steps. In step one, the formylation of *trans*-3,4',5-trimethoxystilbene was achieved with a slight excess of phosphorous oxychloride in dimethylformamide at 0 °C to yield *trans*-2-formyl-3,4',5-trimethoxystilbene. In step two, a mixture of *trans*-2-formyl-3, 4', 5-trimethoxystilbene and quinuclidin-3-one were refluxed in ethanol in the presence of 10% NaOH to yield the title compound, *(Z)*-2-(2,4-dimethoxy-6-(*(E)*-4-methoxystyryl) benzylidene)quinuclidin-3-one in 40% yield.

The X-ray analysis studies revealed that the double bond connecting the quinuclidin-3-one ring and the trimethoxyresveratrol moiety had the *Z* geometry. The dihederal angle between the two phenyl rings in the stilbene skeletone is 32.80 (8)°. The crystal packing is stabilized by van der Waals forces with no intermolecular hydrogen bonding interactions.

### Experimental

A mixture of *trans*-2-formyl-3,4',5-trimethoxystilbene (150 mg, 1 mmol), quinuclidin-3-one (89.44 mg, 1.1 mmol), 10% NaOH and ethanol (10 ml) was refluxed for 5 hrs and completion of reaction was monitored by TLC. The resulting reaction mixture was concentrated to remove ethanol and extracted into ethyl acetate; the ethyl acetate extract washed with water to remove residual NaOH. The organic layer was then dried over anhydrous magnesium sulfate, filtered, and the solvent evaporated to afford the crude product. Purification was achieved by flash silica gel chromatography eluting with 4:1 hexane/ethyl acetate as moblile phase. The title compound, *(Z)*-2-(2,4-dimethoxy-6-(*(E)*-4-methoxystyryl) benzylidene)quinuclidin-3-one, was crystallized from methanol to afford a white crystalline product which was suitable for X-ray analysis. ^1^H NMR (DMSO-d_6_): δ 1.96–1.97 (*d*, *J*=3 Hz, 4H), 2.63 (*s*, 1H), 2.90–2.92 (*m*, *J*=6 Hz, 4H), 3.72–3.82 (*m*, 6H), 3.86 (*s*, 3H), 6.39–6.40 (*d*, *J*=3 Hz, 1H), 6.78–6.79 (*d*, *J*=3 Hz, 1H), 6.86–6.89 (*d*, *J*=9 Hz, 2H), 6.95–6.97 (*d*, *J*=6 Hz, 2H), 7.26 (*s*, 1H), 7.38–7.40 (*d*, *J*=6 Hz, 2H), p.p.m.. ^13^C NMR (DMSO-d_6_): δ 26.1, 41.0, 48.2, 49.7, 55.6, 55.7, 55.9, 97.9, 101.6, 114.3, 123.9, 125.4, 127.9, 128.0, 129.8, 130.2, 138.4, 158.8, 159.5, 160.9. *M*.p: 178–180 °C.

### Refinement

H atoms were found in difference Fourier maps and subsequently placed in idealized positions with constrained distances of 0.98 Å (RCH_3_), 0.99 Å (*R*_2_CH_2_), 1.00 Å (*R*_3_CH), 0.95 Å (C_Ar_H), and with *U*_iso_(H) values set to either 1.2*U*_eq_ or 1.5*U*_eq_ (RCH_3_) of the attached atom.Since this is a light atom structure determined with Mo Kα radiation, there is no anomalous signal with which to refine a meaningful Flack parameter. For this reason, Friedel pairs were merged for the final rounds of refinement.

### Crystal data

**Table:**

C_25_H_27_NO_4_	*F*(000) = 864
*M**_r_* = 405.48	*D*_x_ = 1.279 Mg m^−^^3^
Orthorhombic, *P**c**a*2_1_	Mo *K*α radiation, λ = 0.71073 Å
Hall symbol: P 2c -2ac	Cell parameters from 2806 reflections
*a* = 36.1068 (1) Å	θ = 1.0–27.5°
*b* = 6.8748 (1) Å	µ = 0.09 mm^−^^1^
*c* = 8.4813 (4) Å	*T* = 90 K
*V* = 2105.29 (10) Å^3^	Plate, pale yellow
*Z* = 4	0.26 × 0.20 × 0.08 mm

### Data collection

**Table:**

Nonius KappaCCD diffractometer	2554 independent reflections
Radiation source: fine-focus sealed tube	2313 reflections with *I* > 2σ(*I*)
Graphite monochromator	*R*_int_ = 0.051
Detector resolution: 9.1 pixels mm^-1^	θ_max_ = 27.5°, θ_min_ = 2.3°
ω scans at fixed χ = 55°	*h* = −46→46
Absorption correction: multi-scan (*SCALEPACK*; Otwinowski & Minor, 1997)	*k* = −8→8
*T*_min_ = 0.978, *T*_max_ = 0.993	*l* = −10→11
39211 measured reflections	

### Refinement

**Table:**

Refinement on *F*^2^	Primary atom site location: structure-invariant direct methods
Least-squares matrix: full	Secondary atom site location: difference Fourier map
*R*[*F*^2^ > 2σ(*F*^2^)] = 0.035	Hydrogen site location: inferred from neighbouring sites
*wR*(*F*^2^) = 0.090	H-atom parameters constrained
*S* = 1.04	*w* = 1/[σ^2^(*F*_o_^2^) + (0.055*P*)^2^ + 0.423*P*] where *P* = (*F*_o_^2^ + 2*F*_c_^2^)/3
2554 reflections	(Δ/σ)_max_ < 0.001
274 parameters	Δρ_max_ = 0.17 e Å^−^^3^
1 restraint	Δρ_min_ = −0.19 e Å^−^^3^

### Special details

**Table:**

Geometry. All e.s.d.'s (except the e.s.d. in the dihedral angle between two l.s. planes) are estimated using the full covariance matrix. The cell e.s.d.'s are taken into account individually in the estimation of e.s.d.'s in distances, angles and torsion angles; correlations between e.s.d.'s in cell parameters are only used when they are defined by crystal symmetry. An approximate (isotropic) treatment of cell e.s.d.'s is used for estimating e.s.d.'s involving l.s. planes.
Refinement. Refinement of *F*^2^ against ALL reflections. The weighted *R*-factor *wR* and goodness of fit *S* are based on *F*^2^, conventional *R*-factors *R* are based on *F*, with *F* set to zero for negative *F*^2^. The threshold expression of *F*^2^ > 2σ(*F*^2^) is used only for calculating *R*-factors(gt) *etc*. and is not relevant to the choice of reflections for refinement. *R*-factors based on *F*^2^ are statistically about twice as large as those based on *F*, and *R*- factors based on ALL data will be even larger.

### Fractional atomic coordinates and isotropic or equivalent isotropic displacement parameters (Å^2^)

**Table:**

	*x*	*y*	*z*	*U*_iso_*/*U*_eq_	
C1	0.38605 (5)	0.0394 (3)	−0.0016 (2)	0.0199 (4)	
C2	0.41142 (5)	−0.0668 (3)	−0.0926 (2)	0.0209 (4)	
H2	0.4360	−0.0203	−0.1052	0.025*	
O3	0.42772 (4)	−0.3314 (2)	−0.25036 (18)	0.0242 (3)	
C3	0.40072 (5)	−0.2399 (3)	−0.1643 (2)	0.0208 (4)	
C4	0.36460 (5)	−0.3113 (3)	−0.1488 (2)	0.0213 (4)	
H4	0.3575	−0.4302	−0.1971	0.026*	
O5	0.30344 (4)	−0.2577 (2)	−0.0355 (2)	0.0273 (4)	
C5	0.33950 (5)	−0.2038 (3)	−0.0609 (2)	0.0210 (4)	
C6	0.34939 (5)	−0.0292 (3)	0.0138 (2)	0.0194 (4)	
C7	0.32098 (5)	0.0712 (3)	0.1075 (3)	0.0208 (4)	
H7	0.3007	0.1275	0.0524	0.025*	
C8	0.32148 (5)	0.0892 (3)	0.2640 (3)	0.0213 (4)	
N9	0.34916 (5)	−0.0003 (3)	0.3624 (2)	0.0275 (4)	
C10	0.36272 (6)	0.1427 (4)	0.4797 (3)	0.0343 (5)	
H10A	0.3752	0.2509	0.4239	0.041*	
H10B	0.3812	0.0793	0.5488	0.041*	
C11	0.33104 (6)	0.2257 (4)	0.5823 (3)	0.0323 (5)	
H11A	0.3354	0.1939	0.6946	0.039*	
H11B	0.3301	0.3690	0.5714	0.039*	
C12	0.29427 (6)	0.1356 (4)	0.5273 (3)	0.0312 (5)	
H12	0.2728	0.1886	0.5883	0.037*	
O13	0.26589 (4)	0.2760 (3)	0.2957 (2)	0.0409 (4)	
C13	0.29067 (5)	0.1800 (3)	0.3542 (3)	0.0264 (4)	
C14	0.29709 (7)	−0.0863 (4)	0.5442 (3)	0.0414 (6)	
H14A	0.2742	−0.1482	0.5040	0.050*	
H14B	0.2999	−0.1214	0.6567	0.050*	
C15	0.33094 (7)	−0.1599 (4)	0.4492 (3)	0.0398 (6)	
H15A	0.3490	−0.2202	0.5223	0.048*	
H15B	0.3228	−0.2608	0.3736	0.048*	
C16	0.39711 (5)	0.2215 (3)	0.0766 (2)	0.0199 (4)	
H16	0.3783	0.3145	0.0986	0.024*	
C17	0.43187 (5)	0.2647 (3)	0.1187 (3)	0.0206 (4)	
H17	0.4502	0.1709	0.0933	0.025*	
C18	0.44483 (5)	0.4405 (3)	0.1996 (2)	0.0188 (4)	
C19	0.42275 (5)	0.6030 (3)	0.2311 (2)	0.0207 (4)	
H19	0.3978	0.6041	0.1954	0.025*	
C20	0.43624 (5)	0.7628 (3)	0.3131 (3)	0.0207 (4)	
H20	0.4207	0.8714	0.3332	0.025*	
O21	0.48955 (3)	0.9124 (2)	0.4451 (2)	0.0257 (3)	
C21	0.47287 (5)	0.7621 (3)	0.3656 (2)	0.0201 (4)	
C22	0.49550 (5)	0.6031 (3)	0.3342 (2)	0.0215 (4)	
H22	0.5205	0.6030	0.3693	0.026*	
C23	0.48175 (5)	0.4451 (3)	0.2520 (2)	0.0205 (4)	
H23	0.4975	0.3377	0.2305	0.025*	
C24	0.29163 (6)	−0.4419 (3)	−0.0932 (3)	0.0346 (6)	
H24A	0.2925	−0.4419	−0.2086	0.052*	
H24B	0.2662	−0.4669	−0.0583	0.052*	
H24C	0.3080	−0.5437	−0.0523	0.052*	
C25	0.41767 (6)	−0.5036 (3)	−0.3349 (3)	0.0248 (4)	
H25A	0.4101	−0.6047	−0.2601	0.037*	
H25B	0.4390	−0.5497	−0.3960	0.037*	
H25C	0.3971	−0.4745	−0.4064	0.037*	
C26	0.46609 (6)	1.0558 (3)	0.5147 (3)	0.0263 (4)	
H26A	0.4526	1.1251	0.4317	0.039*	
H26B	0.4812	1.1484	0.5747	0.039*	
H26C	0.4484	0.9923	0.5856	0.039*	

### Atomic displacement parameters (Å^2^)

**Table:**

	*U*^11^	*U*^22^	*U*^33^	*U*^12^	*U*^13^	*U*^23^
C1	0.0212 (9)	0.0204 (9)	0.0179 (9)	0.0000 (7)	−0.0011 (8)	−0.0002 (8)
C2	0.0177 (8)	0.0241 (9)	0.0209 (10)	−0.0021 (7)	0.0001 (7)	−0.0010 (8)
O3	0.0200 (6)	0.0251 (7)	0.0275 (8)	−0.0008 (5)	0.0031 (6)	−0.0089 (6)
C3	0.0211 (9)	0.0230 (9)	0.0184 (10)	0.0023 (7)	0.0002 (8)	−0.0020 (8)
C4	0.0218 (9)	0.0199 (9)	0.0222 (10)	−0.0010 (7)	−0.0013 (8)	−0.0037 (8)
O5	0.0195 (6)	0.0257 (7)	0.0367 (9)	−0.0073 (6)	0.0054 (6)	−0.0102 (7)
C5	0.0186 (8)	0.0233 (9)	0.0210 (10)	−0.0021 (7)	−0.0002 (8)	−0.0020 (8)
C6	0.0197 (9)	0.0202 (9)	0.0185 (9)	−0.0008 (7)	−0.0008 (7)	−0.0007 (8)
C7	0.0184 (8)	0.0192 (9)	0.0247 (10)	−0.0001 (7)	−0.0029 (8)	−0.0018 (9)
C8	0.0169 (8)	0.0207 (9)	0.0261 (10)	0.0024 (7)	−0.0010 (8)	−0.0039 (8)
N9	0.0258 (8)	0.0354 (10)	0.0213 (8)	0.0083 (8)	−0.0045 (8)	−0.0037 (8)
C10	0.0235 (10)	0.0549 (15)	0.0245 (11)	0.0018 (10)	−0.0039 (9)	−0.0091 (11)
C11	0.0255 (10)	0.0474 (13)	0.0242 (11)	0.0016 (9)	−0.0036 (9)	−0.0080 (11)
C12	0.0187 (9)	0.0482 (13)	0.0268 (11)	0.0008 (9)	0.0011 (8)	−0.0106 (11)
O13	0.0304 (8)	0.0562 (11)	0.0363 (9)	0.0215 (8)	−0.0095 (7)	−0.0178 (9)
C13	0.0197 (9)	0.0300 (10)	0.0296 (11)	0.0041 (8)	−0.0040 (9)	−0.0113 (10)
C14	0.0387 (12)	0.0516 (15)	0.0338 (13)	−0.0093 (11)	0.0032 (11)	0.0046 (12)
C15	0.0522 (14)	0.0343 (12)	0.0329 (13)	0.0067 (11)	−0.0044 (12)	0.0053 (11)
C16	0.0214 (9)	0.0202 (9)	0.0182 (9)	−0.0004 (7)	0.0014 (8)	−0.0013 (8)
C17	0.0199 (8)	0.0204 (9)	0.0214 (10)	0.0002 (7)	0.0004 (8)	−0.0002 (8)
C18	0.0188 (8)	0.0201 (9)	0.0174 (9)	−0.0023 (7)	0.0019 (7)	−0.0007 (8)
C19	0.0196 (8)	0.0226 (10)	0.0199 (10)	−0.0015 (7)	−0.0019 (7)	0.0003 (8)
C20	0.0202 (8)	0.0198 (9)	0.0221 (10)	0.0020 (7)	0.0003 (8)	−0.0011 (8)
O21	0.0209 (6)	0.0221 (7)	0.0342 (8)	−0.0006 (5)	−0.0030 (6)	−0.0099 (7)
C21	0.0216 (9)	0.0193 (9)	0.0193 (9)	−0.0041 (7)	−0.0008 (8)	−0.0027 (8)
C22	0.0173 (8)	0.0241 (9)	0.0230 (10)	−0.0013 (7)	−0.0013 (8)	0.0001 (9)
C23	0.0192 (8)	0.0203 (9)	0.0220 (9)	0.0008 (7)	0.0015 (8)	−0.0020 (8)
C24	0.0294 (10)	0.0271 (11)	0.0473 (15)	−0.0112 (8)	0.0071 (10)	−0.0119 (11)
C25	0.0264 (9)	0.0228 (10)	0.0251 (11)	0.0009 (8)	−0.0007 (9)	−0.0072 (9)
C26	0.0291 (10)	0.0222 (10)	0.0275 (11)	0.0026 (8)	−0.0022 (9)	−0.0069 (9)

### Geometric parameters (Å, º)

**Table:**

C1—C2	1.403 (3)	C14—C15	1.549 (4)
C1—C6	1.411 (3)	C14—H14A	0.9900
C1—C16	1.472 (3)	C14—H14B	0.9900
C2—C3	1.391 (3)	C15—H15A	0.9900
C2—H2	0.9500	C15—H15B	0.9900
O3—C3	1.371 (2)	C16—C17	1.338 (3)
O3—C25	1.431 (2)	C16—H16	0.9500
C3—C4	1.400 (3)	C17—C18	1.466 (3)
C4—C5	1.387 (3)	C17—H17	0.9500
C4—H4	0.9500	C18—C19	1.398 (3)
O5—C5	1.371 (2)	C18—C23	1.405 (3)
O5—C24	1.422 (2)	C19—C20	1.388 (3)
C5—C6	1.403 (3)	C19—H19	0.9500
C6—C7	1.470 (3)	C20—C21	1.395 (3)
C7—C8	1.333 (3)	C20—H20	0.9500
C7—H7	0.9500	O21—C21	1.373 (2)
C8—N9	1.440 (3)	O21—C26	1.428 (2)
C8—C13	1.488 (3)	C21—C22	1.391 (3)
N9—C15	1.476 (3)	C22—C23	1.383 (3)
N9—C10	1.482 (3)	C22—H22	0.9500
C10—C11	1.546 (3)	C23—H23	0.9500
C10—H10A	0.9900	C24—H24A	0.9800
C10—H10B	0.9900	C24—H24B	0.9800
C11—C12	1.538 (3)	C24—H24C	0.9800
C11—H11A	0.9900	C25—H25A	0.9800
C11—H11B	0.9900	C25—H25B	0.9800
C12—C13	1.505 (3)	C25—H25C	0.9800
C12—C14	1.536 (4)	C26—H26A	0.9800
C12—H12	1.0000	C26—H26B	0.9800
O13—C13	1.217 (3)	C26—H26C	0.9800
			
C2—C1—C6	119.32 (18)	C12—C14—H14B	109.8
C2—C1—C16	120.87 (16)	C15—C14—H14B	109.8
C6—C1—C16	119.81 (17)	H14A—C14—H14B	108.3
C3—C2—C1	120.27 (17)	N9—C15—C14	111.6 (2)
C3—C2—H2	119.9	N9—C15—H15A	109.3
C1—C2—H2	119.9	C14—C15—H15A	109.3
C3—O3—C25	117.78 (15)	N9—C15—H15B	109.3
O3—C3—C2	115.32 (16)	C14—C15—H15B	109.3
O3—C3—C4	123.48 (17)	H15A—C15—H15B	108.0
C2—C3—C4	121.20 (17)	C17—C16—C1	124.32 (17)
C5—C4—C3	118.17 (17)	C17—C16—H16	117.8
C5—C4—H4	120.9	C1—C16—H16	117.8
C3—C4—H4	120.9	C16—C17—C18	127.40 (17)
C5—O5—C24	118.14 (16)	C16—C17—H17	116.3
O5—C5—C4	124.11 (17)	C18—C17—H17	116.3
O5—C5—C6	113.72 (17)	C19—C18—C23	117.55 (17)
C4—C5—C6	122.16 (17)	C19—C18—C17	124.46 (16)
C5—C6—C1	118.86 (17)	C23—C18—C17	117.98 (16)
C5—C6—C7	117.92 (16)	C20—C19—C18	121.85 (17)
C1—C6—C7	123.20 (18)	C20—C19—H19	119.1
C8—C7—C6	124.95 (19)	C18—C19—H19	119.1
C8—C7—H7	117.5	C19—C20—C21	119.34 (18)
C6—C7—H7	117.5	C19—C20—H20	120.3
C7—C8—N9	123.15 (19)	C21—C20—H20	120.3
C7—C8—C13	122.75 (19)	C21—O21—C26	117.55 (14)
N9—C8—C13	113.58 (18)	O21—C21—C22	115.33 (16)
C8—N9—C15	107.31 (17)	O21—C21—C20	124.75 (17)
C8—N9—C10	109.56 (17)	C22—C21—C20	119.89 (17)
C15—N9—C10	107.79 (18)	C23—C22—C21	120.20 (17)
N9—C10—C11	112.23 (17)	C23—C22—H22	119.9
N9—C10—H10A	109.2	C21—C22—H22	119.9
C11—C10—H10A	109.2	C22—C23—C18	121.16 (18)
N9—C10—H10B	109.2	C22—C23—H23	119.4
C11—C10—H10B	109.2	C18—C23—H23	119.4
H10A—C10—H10B	107.9	O5—C24—H24A	109.5
C12—C11—C10	108.62 (18)	O5—C24—H24B	109.5
C12—C11—H11A	110.0	H24A—C24—H24B	109.5
C10—C11—H11A	110.0	O5—C24—H24C	109.5
C12—C11—H11B	110.0	H24A—C24—H24C	109.5
C10—C11—H11B	110.0	H24B—C24—H24C	109.5
H11A—C11—H11B	108.3	O3—C25—H25A	109.5
C13—C12—C14	107.4 (2)	O3—C25—H25B	109.5
C13—C12—C11	106.79 (19)	H25A—C25—H25B	109.5
C14—C12—C11	108.32 (19)	O3—C25—H25C	109.5
C13—C12—H12	111.4	H25A—C25—H25C	109.5
C14—C12—H12	111.4	H25B—C25—H25C	109.5
C11—C12—H12	111.4	O21—C26—H26A	109.5
O13—C13—C8	124.6 (2)	O21—C26—H26B	109.5
O13—C13—C12	124.8 (2)	H26A—C26—H26B	109.5
C8—C13—C12	110.61 (18)	O21—C26—H26C	109.5
C12—C14—C15	109.2 (2)	H26A—C26—H26C	109.5
C12—C14—H14A	109.8	H26B—C26—H26C	109.5
C15—C14—H14A	109.8		
			
C6—C1—C2—C3	−1.2 (3)	C10—C11—C12—C14	59.0 (3)
C16—C1—C2—C3	179.23 (18)	C7—C8—C13—O13	−13.8 (3)
C25—O3—C3—C2	−176.17 (18)	N9—C8—C13—O13	174.2 (2)
C25—O3—C3—C4	3.3 (3)	C7—C8—C13—C12	165.8 (2)
C1—C2—C3—O3	179.94 (18)	N9—C8—C13—C12	−6.2 (3)
C1—C2—C3—C4	0.5 (3)	C14—C12—C13—O13	125.5 (2)
O3—C3—C4—C5	−178.81 (19)	C11—C12—C13—O13	−118.5 (2)
C2—C3—C4—C5	0.6 (3)	C14—C12—C13—C8	−54.1 (2)
C24—O5—C5—C4	4.7 (3)	C11—C12—C13—C8	61.9 (2)
C24—O5—C5—C6	−174.0 (2)	C13—C12—C14—C15	58.5 (3)
C3—C4—C5—O5	−179.51 (19)	C11—C12—C14—C15	−56.5 (3)
C3—C4—C5—C6	−1.0 (3)	C8—N9—C15—C14	−56.1 (3)
O5—C5—C6—C1	178.92 (18)	C10—N9—C15—C14	61.8 (2)
C4—C5—C6—C1	0.2 (3)	C12—C14—C15—N9	−3.7 (3)
O5—C5—C6—C7	0.2 (3)	C2—C1—C16—C17	−25.6 (3)
C4—C5—C6—C7	−178.45 (19)	C6—C1—C16—C17	154.9 (2)
C2—C1—C6—C5	0.9 (3)	C1—C16—C17—C18	−178.61 (19)
C16—C1—C6—C5	−179.58 (18)	C16—C17—C18—C19	−7.2 (3)
C2—C1—C6—C7	179.48 (19)	C16—C17—C18—C23	171.5 (2)
C16—C1—C6—C7	−1.0 (3)	C23—C18—C19—C20	−0.9 (3)
C5—C6—C7—C8	111.4 (2)	C17—C18—C19—C20	177.7 (2)
C1—C6—C7—C8	−67.3 (3)	C18—C19—C20—C21	0.1 (3)
C6—C7—C8—N9	−4.3 (4)	C26—O21—C21—C22	−164.65 (18)
C6—C7—C8—C13	−175.52 (18)	C26—O21—C21—C20	17.1 (3)
C7—C8—N9—C15	−108.9 (2)	C19—C20—C21—O21	178.75 (19)
C13—C8—N9—C15	63.0 (2)	C19—C20—C21—C22	0.6 (3)
C7—C8—N9—C10	134.3 (2)	O21—C21—C22—C23	−178.73 (18)
C13—C8—N9—C10	−53.7 (2)	C20—C21—C22—C23	−0.4 (3)
C8—N9—C10—C11	57.3 (2)	C21—C22—C23—C18	−0.5 (3)
C15—N9—C10—C11	−59.2 (2)	C19—C18—C23—C22	1.1 (3)
N9—C10—C11—C12	−1.3 (3)	C17—C18—C23—C22	−177.61 (19)
C10—C11—C12—C13	−56.4 (2)		

## References

[bb1] Aggarwal, B. B., Bhardwaj, A., Aggarwal, R. S., Seeram, N. P., Shishodia, S. & Takada, Y. (2004). *Anticancer Res.* **24**, 2783–2840. 15517885

[bb2] Cushman, M., Nagarathnam, D., Gopal, D., Chakraborti, A. K., Lin, C. M. & Hamel, E. (1991). *J. Med. Chem.* **34**, 2579–2588. 10.1021/jm00112a036 1875350

[bb3] Jeandet, P., Bessis, R., Maume, B. F., Meunier, P., Peyron, D. & Trollat, P. J. (1979). *J. Agric. Food Chem.* **27**, 984–989.

[bb4] Nonius (1998). *COLLECT* Nonius BV, Delft, The Netherlands.

[bb5] Otwinowski, Z. & Minor, W. (1997). *Methods in Enzymology*, Vol. 276, *Macromolecular Crystallography*, Part A, edited by C. W. Carter Jr & R. M. Sweet, pp. 307–326. New York: Academic Press.

[bb6] Pettit, G. R., Singh, S. B., Boyd, M. R., Hamel, E., Pettit, R. K., Schmidt, J. M. & Hogan, F. (1995). *J. Med. Chem.* **38**, 1666–1672. 10.1021/jm00010a011 7752190

[bb7] Sheldrick, G. M. (2008). *Acta Cryst.* A**64**, 112–122. 10.1107/S0108767307043930 18156677

[bb8] Trela, B. C. & Waterhouse, A. L. (1996). *J. Agric. Food Chem.* **44**, 1253–1257.

